# Impact of Antipsychotic Medication on Metabolic Syndrome in Psychiatry

**DOI:** 10.1192/j.eurpsy.2026.11382

**Published:** 2026-07-31

**Authors:** S. Aouadi, A. Aissa, Y. Ben Othman, A. Hlaoui, R. Hosni, R. Jomli

**Affiliations:** 1 Avicenne Psychiatry; 2 Psychiatry, Hospital Razi, Tunis, Tunisia

## Abstract

**Introduction:**

Antipsychotic medications, while essential for the management of psychiatric disorders, are associated with significant metabolic adverse effects that can compromise overall health. Among these, **metabolic syndrome** represents a major clinical concern due to its established link with **increased cardiovascular morbidity and mortality**.

**Objectives:**

To determine the **prevalence of metabolic syndrome** among patients receiving antipsychotic treatment and to analyze the **correlations between different classes of antipsychotics** and the occurrence of this syndrome.

**Methods:**

A **cross-sectional observational study** was conducted in the Psychiatry Department of Avicenne, El Razi Psychiatric Hospital in Tunis, from **January 2025 to April 2025**. Clinically stable outpatients under long-term antipsychotic treatment were included. Data were collected using a standardized clinical record form, and current antipsychotic regimens were retrieved from patient medical files.

**Metabolic syndrome** was defined according to the **National Cholesterol Education Program Adult Treatment Panel III (NCEP ATP III)** criteria. Statistical analyses were performed using **IBM SPSS version 25**.

**Results:**

Evaluation of metabolic syndrome prevalence according to the NCEP ATP III criteria revealed a **high rate of metabolic comorbidities** among patients treated with antipsychotics.

Among the **31 patients included**, **77.8%** had a **family history of type 2 diabetes mellitus**, and **88.9%** had a **family history of hypertension**, both being major risk factors for metabolic syndrome.

Regarding treatment distribution, **22.2%** of patients were receiving **haloperidol**, **14.8% fluphenazine decanoate**, **22.2%risperidone**, **37% olanzapine**, **25.9% clozapine**, and **96.3% quetiapine**.

Statistical analysis demonstrated a **significant association** between the occurrence of metabolic syndrome and exposure to **olanzapine (p = 0.041)** and **clozapine (p = 0.03)**, indicating a more pronounced disturbance of **metabolic homeostasis**with these agents. Conversely, **no significant correlation** was found with **haloperidol, risperidone, or quetiapine**. The absence of significance for these agents may be attributed to the **heterogeneous distribution of treatments** within the study sample.

**Conclusions:**

This study highlights a **significant association** between exposure to certain **atypical antipsychotics**, particularly **olanzapine and clozapine**, and the development of **metabolic syndrome**. These findings emphasize the importance of **systematic screening** and **early management of metabolic abnormalities** to mitigate **cardiometabolic complications**associated with antipsychotic therapy.

An **individualized therapeutic approach**, taking into account each patient’s **metabolic risk profile**, is essential to optimize the **benefit–risk balance** of antipsychotic medications and to improve long-term psychiatric care outcomes.

**Disclosure of Interest:**

None Declared

